# Concentration-Dependent Pore Morphology in Cellulose Acetate Coatings for Enhanced Radiative Cooling

**DOI:** 10.3390/ma19194255

**Published:** 2026-10-08

**Authors:** Haopeng Yu, Rui Yang, Xiaohua Feng, Pengyu Li, Hua Li

**Affiliations:** 1 Henan Institute of Advanced Technology, Zhengzhou University, Zhengzhou 450003, China; yuhaopeng0906@163.com; 2 State Key Laboratory of Advanced Marine Materials, Zhejiang-Japan Joint Laboratory for Antibacterial and Antifouling Technology and Zhejiang Engineering Research Center for Biomedical Materials, Ningbo Institute of Materials Technology and Engineering, Chinese Academy of Sciences, Ningbo 315201, China; yangrui@nimte.ac.cn (R.Y.); lipengyu@nimte.ac.cn (P.L.)

**Keywords:** cellulose acetate coatings, non-solvent-induced phase separation method, radiative cooling performance, porosity structures

## Abstract

This study employs the non-solvent-induced phase separation (NIPS) method to fabricate porous cellulose acetate (CA) coatings. By optimizing the concentration of CA and *N*, *N*-dimethylacetamide (DMAC), the pore structure and size of the coatings are effectively controlled. The influence of homogeneous solution concentration on the optical properties of CA coatings is explored, and the relationship between the microstructure of the coatings and their radiative cooling performance is analyzed in depth. A self-assembled radiative cooling device is used to experimentally evaluate the cooling effect of the porous CA coatings. The results show that when the CA concentration reaches 15 wt%, the coating exhibits an ordered transition from irregular large pores to well-defined finger-like pores and then to sponge-like pores, achieving the optimal radiative cooling performance. At this concentration, the coating achieves a high solar reflectance (0.3–2.5 μm) of 96.56% and an emissivity of 0.96 in the atmospheric transparency window (8–13 μm). Compared to the uncoated substrate, the temperature difference reaches 18.88 °C, and the cooling effect relative to the ambient temperature is 2.6 °C, demonstrating excellent radiative cooling performance. After 10 cycles of the damp–heat cyclic test, the optical properties of the coating showed almost no change, indicating excellent damp–heat resistance.

## 1. Introduction

All-weather radiative cooling technology leverages the radiative heat dissipation properties of materials to achieve continuous cooling under diverse weather conditions, operating effectively both during the day and at night. The fundamental principle is to design radiative cooling materials that efficiently emit thermal radiation within the atmospheric transparency window (8–13 μm) while exhibiting low absorption in the visible and near-infrared spectra to minimize solar heating [1,2,3,4,5].

To enhance all-weather cooling performance, researchers typically employ strategies such as tuning material micro/nanostructures [6,7], optimizing spectral selectivity [8], designing composite material [9,10] for porous structure [11,12,13], adding metal–dielectric composite material [14,15,16], or depositing highly reflective coatings [17,18], which can be utilized to enhance solar reflection and improve thermal radiation efficiency. Organic polymer materials are commonly used in this field due to their lightweight nature, ease of processing, and excellent infrared radiation properties. Commonly used organic materials include polydimethylsiloxane (PDMS) [19,20], poly (vinylidene fluoride) (PVDF) [21,22], and their composites [23,24,25]. However, the high transparency of PDMS within the visible spectrum fundamentally restricts its daytime radiative cooling performance when employed in standalone configurations. While PVDF exhibits a high reflectance, it contains F elements, which raises concerns regarding its environmental sustainability. Cellulose acetate (CA) exhibits high reflectivity across the entire solar spectrum [26] while also achieving high emissivity in the atmospheric transparency window due to the vibrational absorption of its functional groups [27]. Additionally, CA is biodegradable and environmentally friendly [28], making it a promising material for applications in radiative cooling.

In addition to the intrinsic optical properties of the material, the structure of the coating, particularly the shape and size of the pores within the coating [29,30], can also significantly impact the radiative cooling performance. The non-solvent-induced phase separation method (NIPS) allows for the control of the solvent type, concentration, and deposition conditions to regulate the shape and size of the pores in the coating [31,32]. This enables the fabrication of coating materials with high porosity structures, optimizing the surface architecture and enhancing the optical properties of the coating materials. This method can be applied to the preparation of porous structure coatings. However, systematic studies on the preparation of different microporous structures via the NIPS method for optical performance modulation are relatively scarce. In this work, CA coatings with various microporous structures are fabricated by controlling the concentration of CA/N, N-dimethylacetamide (DMAC) homogeneous solutions. The influence of microporous structures on the radiative cooling performance and the environmental adaptability of the coatings was then investigated, thereby obtaining the optimal process parameters. This study provides new insights for developing green, highly efficient, low-cost, and environmentally tolerant organic polymer radiative cooling materials.

## 2. Experimental Section

CA (acetyl content 39.8 wt%, hydroxyl content 3.5 wt%) was purchased from Shanghai Aladdin Biochemical Technology Co., Ltd., Shanghai, China. DMAC (AR 99%) was purchased from Shanghai Macklin Biochemical Co., Ltd., Shanghai, China. Deionized water was self-prepared in the laboratory.

Totals of 10 g, 15 g and 20 g of CA powder were gradually added to 90 g, 85 g, and 80 g of DMAC, respectively, and stirred magnetically for 3 h until completely dissolved. The solution was then left to stand for 12 h to remove bubbles, resulting in transparent and homogeneous CA/DMAC solutions with concentrations of 10 wt%, 15 wt%, and 20 wt%. The homogeneous CA/DMAC solutions of different concentrations were applied onto sandblasted Al substrates using a scraper. After standing for 10 min, the coated substrates were immersed in deionized water. After 6 h of phase separation, the samples were removed and air-dried at room temperature to obtain CA coatings. The coatings prepared from the 10 wt%, 15 wt%, and 20 wt% CA/DMAC solutions were named 10CA, 15CA, and 20CA, respectively.

The evolution of crystal structure during the preparation of CA coatings was characterized by X-ray diffraction (XRD) at a scanning rate of 6°/min over a 2θ range of 5–80°. The bonding characteristics of the CA coatings were analyzed using a Fourier transform infrared (FTIR) spectrometer (Thermo Fisher IS 50) (Thermo Fisher Scientific, Waltham, MA, USA) in the wavenumber range of 4000–400 cm^−1^. The emissivity of the coatings in the atmospheric transparency window (8–13 μm) was examined using the integrating sphere mode, and the emissivity values were calculated using Kirchhoff’s law from the data obtained by FTIR spectroscopy. The microstructure of the coatings was analyzed using a scanning electron microscope (SEM, Hitachi Regulus 8230, Hitachi High-Technologies Corporation, Tokyo, Japan). The solar reflectance (0.3–2.5 μm) was measured using an ultraviolet–visible–near-infrared (UV-Vis-NIR) spectrophotometer (Agilent Cary 5000, Agilent Technologies, Inc., Santa Clara, CA, USA) in the integrating sphere mode. The radiative cooling performance of the coatings was evaluated using a custom-built outdoor testing setup. As shown in Figure 4a, during the experiment, the uncoated reference sample and the coated sample were separately placed inside a polystyrene foam box, with thermocouples attached to their bottoms to monitor the substrate temperature changes in real time. To minimize the heating effect of solar radiation on the setup, the exterior of the polystyrene foam box was wrapped with an aluminum foil layer to reflect sunlight. Meanwhile, the top of the device was sealed with a high-density polyethylene film to effectively reduce the interference of air convection on the test results. The test condition was as follows: a 2 h outdoor cooling experiment on the radiative cooling coating was carried out at the Cixi, China (30°11′31″ N, 121°17′24″ E), from 13:00 to 15:00 on 3 December 2024. The experiment was conducted under clear-sky conditions, with an ambient temperature of 10–15 °C and a relative humidity of 75%.

## 3. Results and Discussion

**Figure 1:**
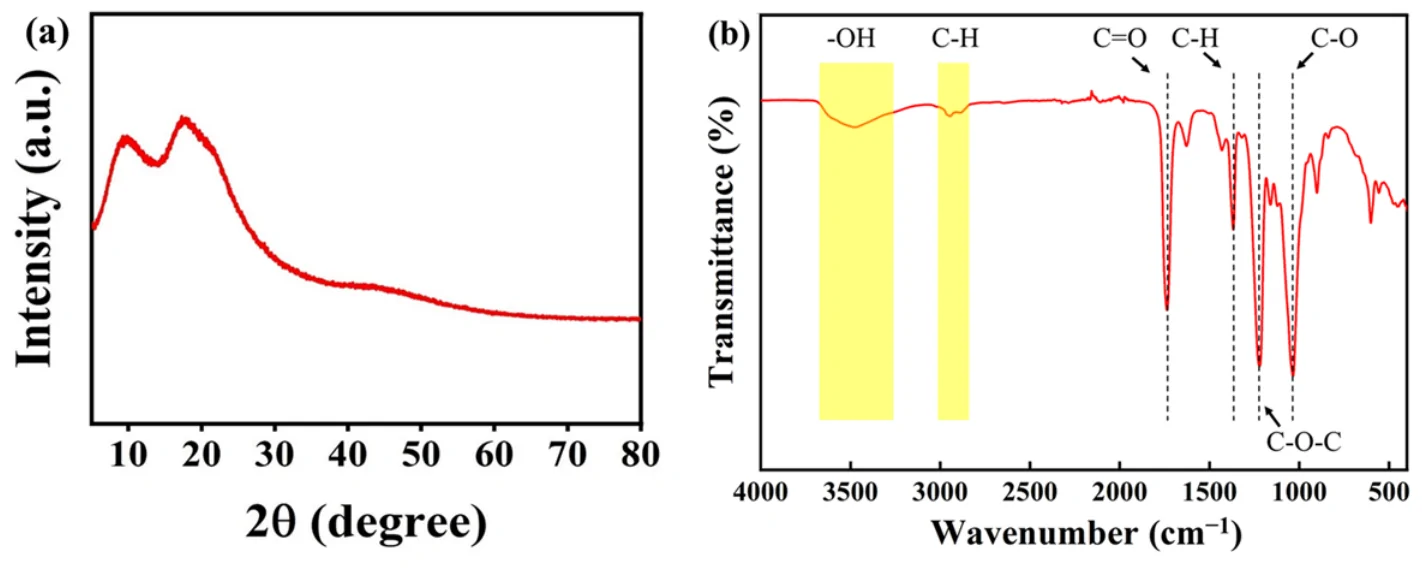
(**a**) XRD and (**b**) infrared spectrum of CA coating.

As observed in the XRD pattern of the CA coating, two broad peaks appear at 2θ = 9.47° and 17.69°, indicating that cellulose acetate predominantly exists in an amorphous state or exhibits low crystallinity (Figure 1a). This phenomenon is closely related to the acetylation process of cellulose acetate: during the acetylation reaction, the hydroxyl groups (-OH) on the cellulose molecular chains are substituted by acetyl groups (-COCH_3_), destroying the original hydrogen bonding network and leading to the disordering of the molecular chain arrangement, thereby significantly reducing the crystallinity of the material.

The CA coating is prepared though the NIPS method. From the infrared spectrum, the broad peak appearing in the range of 3000–3600 cm^−1^ is assigned to the stretching vibration of hydroxyl groups (-OH); since the cellulose acetate used in the experiment is a highly acetylated product with a relatively low number of hydroxyl groups, the intensity of this peak is weak. The absorption peaks in the range of 2800–3000 cm^−1^ correspond to the stretching vibrations of C-H bonds in methyl and methylene groups. The characteristic peak at 1738 cm^−1^ is assigned to the stretching vibration of carbonyl groups (C=O); the peak at 1367 cm^−1^ corresponds to the stretching vibration of saturated C-H bonds; the peak at 1220 cm^−1^ is assigned to the asymmetric stretching vibration of C-O-C bonds; and the peak at 1035 cm^−1^ corresponds to the stretching vibration of the C-O skeleton [33] (Figure 1b). By comparing the infrared spectra of the CA powder and the prepared CA coating, it can be observed that the two spectra are essentially identical. This indicates that during the preparation of the CA coating via the NIPS method, the molecular structure of CA remained undamaged. The formation of the coating is a physical process, confirming the successful preparation of the CA coating.

**Figure 2:**
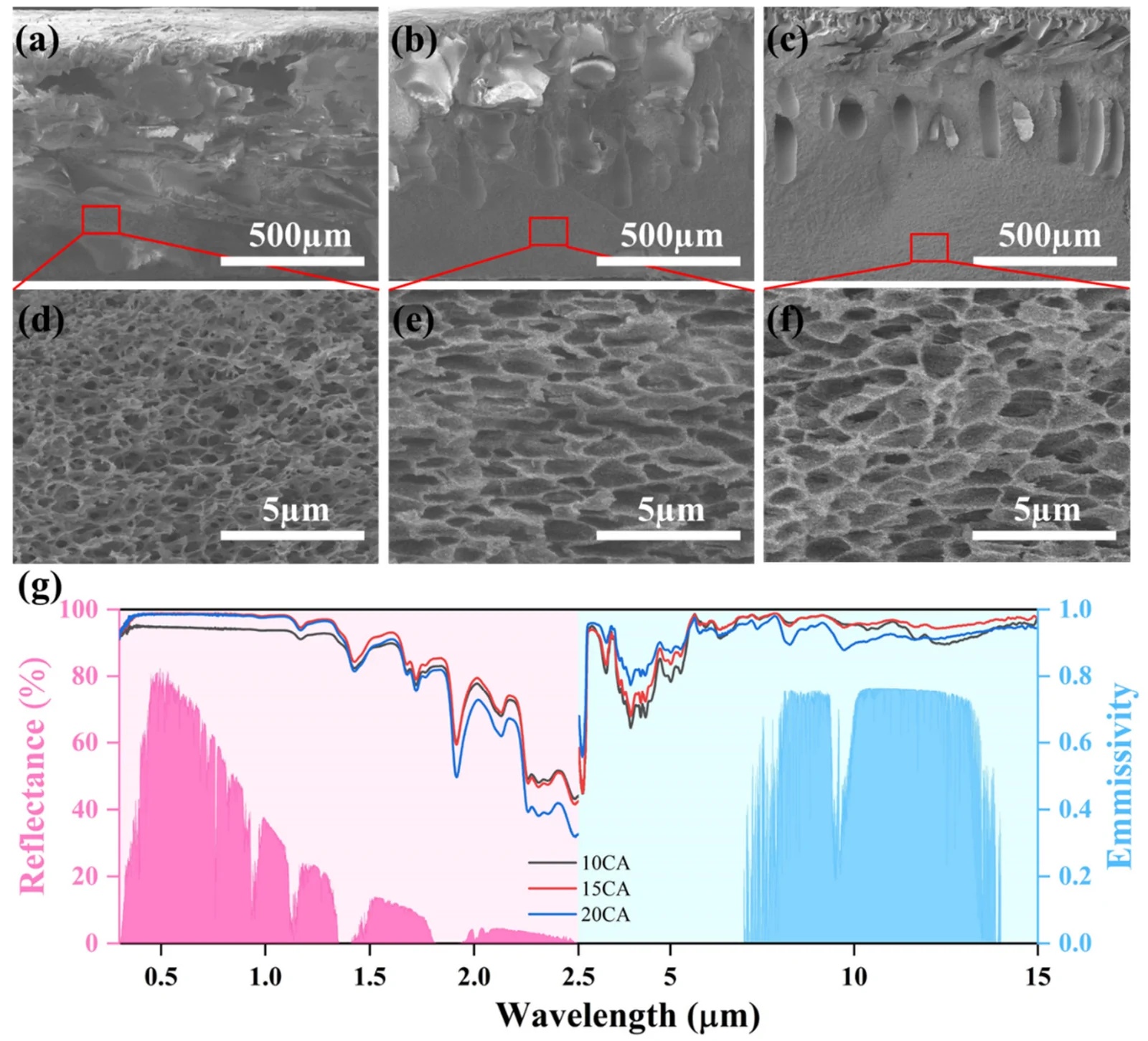
(**a**–**f**) SEM images of the 10CA, 15CA, and 20CA coatings (magnified 100 and 10,000 times). (**g**) the solar reflectance in the 0.3–2.5 µm wavelength range and the emissivity in the 2.5–15 µm range for the 10CA, 15CA, and 20CA coatings.

The cross-sectional morphology of the coatings varies with different CA concentrations. From the surface layer to the inner layer, the structure transitions from nanopores to micropores and then to a spongy micro–nanoporous structure (Figure 2a–c). The 10CA coating exhibits large irregular micropores, with pore walls connected by a spongy structure (Figure 2a). In the 15CA coating, both large irregular pores and finger-like pores appear (Figure 2b). In contrast, the 20CA coating shows almost no irregular pores, with well-defined finger-like pores becoming predominant (Figure 2c). This phenomenon is attributed to the rapid interdiffusion of the solvent and nonsolvent at the interface, leading to transient phase separation. As a result, the high-polymer-concentration regions solidify quickly, forming a nanoporous surface layer. As the nonsolvent penetrates the homogeneous solution and continues to diffuse inward, a concentration gradient is established, and the phase separation process persists. Due to the instability of the phase separation interface, uneven solvent diffusion occurs, forming polymer-lean phase channels (where phase separation occurs rapidly) and a polymer-rich phase matrix (where phase separation occurs more slowly), ultimately leading to the formation of finger-like pores. When the nonsolvent reaches the bottom layer, its penetration speed decreases, and solvent diffusion slows down. This results in delayed phase separation for an extended period, allowing for a higher polymer concentration. Consequently, polymer chains have more time to rearrange, forming a sponge-like porous structure with smaller and more uniformly sized pores [34]. When the CA concentration is 10%, the DMAC content is the highest, resulting in a lower solution viscosity. The rapid interdiffusion between DMAC and water accelerates the phase separation process, making it more intense. Due to the lower polymer content within the coating, the polymer network has insufficient mechanical strength to sustain the stable growth of finger-like pores. As a result, these pores are prone to collapse or merge into irregular large pores [35].

With an increase in the concentration of the homogeneous solution, the solution viscosity rises, slowing down the interdiffusion rate between the solvent and nonsolvent. As a result, the polymer content within the coating increases, providing sufficient structural support for the formation of stable finger-like pores.

The sponge-like pore structures of the three coatings are similar, with pore sizes mainly ranging from 1.0 to 2.5 μm (Figure 2e,f). However, the pore size distribution of 10CA is the widest, and it has the highest porosity, followed by 15CA, while 20CA has the lowest porosity. This is because, as the concentration of the homogeneous solution increases, the viscosity of the solution also increases. Consequently, the phase separation rate at the bottom slows down, leading to a reduction in porosity.

The reflectance and emissivity of the 10CA coating are 92.65% and 0.94, respectively, while those of the 15CA coating are 96.56% and 0.96, and those of the 20CA coating are 95.89% and 0.92 (Figure 2g). Among the three coatings, the 15CA coating exhibits the best optical performance and the most effective radiative cooling capability.

Microstructural analysis of the coatings reveals that the lower reflectance of the 10CA coating can be attributed to the presence of large irregular pores. These pores trap part of the incident sunlight, preventing it from being reflected. When the pore size aligns with the 0.3–2.5 µm wavelength range, Mie scattering is enhanced [36]. The slight decrease in reflectance observed in the 20CA coating may be due to its reduced porosity. Regarding emissivity, in the 8–13 µm atmospheric transparency window, the 10CA coating exhibits a lower emissivity than the 15CA coating but a higher emissivity than the 20CA coating. The emissivity of the 20CA coating is slightly lower than that of the 15CA coating (Figure 2g). This can be attributed to the combined effects of porosity [37] and surface roughness [38]. Emissivity is influenced by multiple factors, including porosity, pore morphology, surface roughness, and coating microstructure. In general, higher porosity tends to reduce emissivity, whereas increased surface roughness may enhance it. For the 10CA coating, although its relatively high porosity could potentially lower emissivity, the pronounced surface roughness appears to compensate for this effect, contributing to its higher emissivity. In contrast, the 20CA coating exhibits lower porosity but a comparatively smoother surface, which may limit its emissivity. The 15CA coating achieves a favorable balance among these competing factors, resulting in superior optical performance and radiative cooling efficiency.

**Figure 3:**
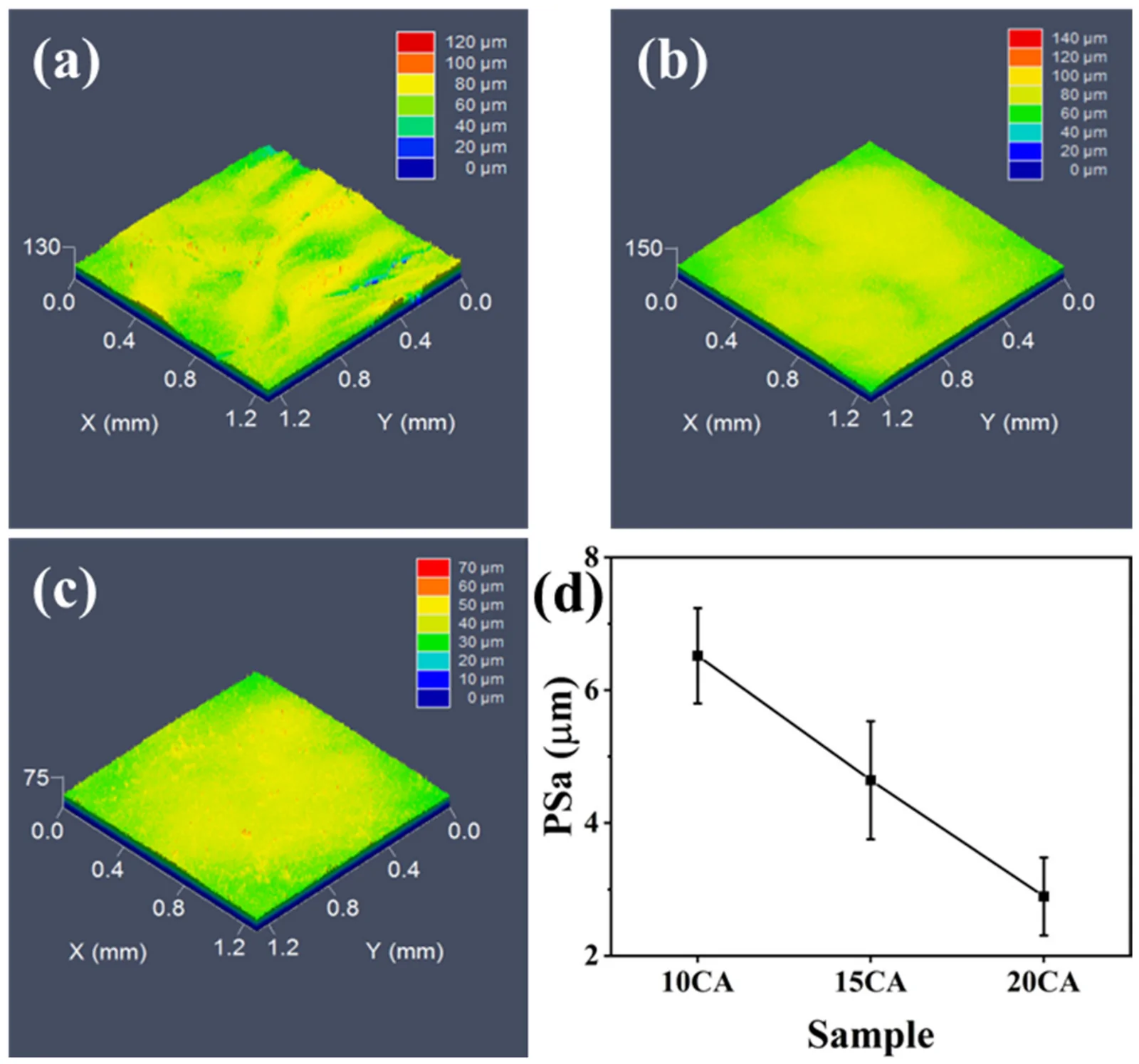
Surface roughness of the coating at different concentrations: (**a**) 10CA, (**b**) 15CA, (**c**) 20CA, (**d**) concentration–roughness curve.

Figure 3 presents the 3D surface topography images and the corresponding roughness variation curves of the coatings prepared at different concentrations. The average Sa values of the 10CA, 15CA, and 20CA coatings are 6.52, 4.64, and 2.89 μm, respectively. The results indicate that the relatively high porosity of the 10CA coating increases the surface roughness, thereby enhancing the emissivity. The 15CA coating achieves the highest emissivity. However, when the concentration is further increased to 20CA, the porosity decreases and the surface becomes relatively smooth, leading to a reduction in emissivity. Therefore, the 15CA coating achieves an optimal balance between porosity and surface roughness, exhibiting the best radiative performance.

**Figure 4:**
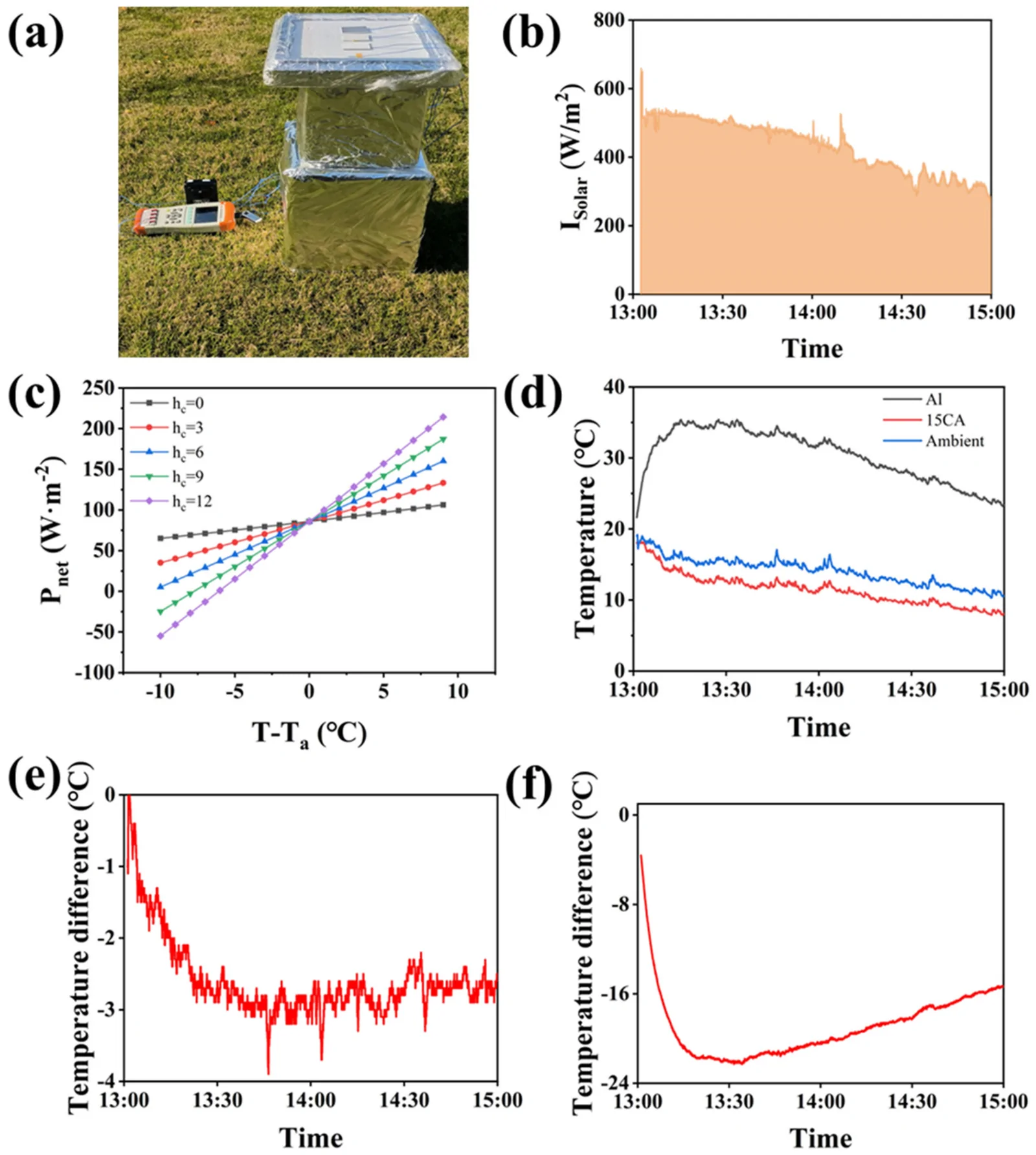
(**a**) Photograph of the outdoor cooling device; (**b**) variation in solar irradiance during the outdoor cooling test; (**c**) theoretical net cooling power; (**d**) temperature variation observed in the outdoor cooling test; (**e**) temperature difference between the 15CA-coated Al substrate and the surrounding environment; (**f**) temperature difference between the 15CA-coated Al substrate and the uncoated Al substrate.

To further validate the cooling performance of the 15CA coating, we conducted an outdoor real-world cooling test using a self-built cooling setup (Figure 4a). An uncoated aluminum plate with dimensions of 40 × 40 × 2 mm was used as a reference. The aluminum plate coated with 15CA and the uncoated aluminum plate were placed inside a polystyrene foam box, with thermocouples attached at the bottom. The exterior of the box was wrapped with aluminum foil to reduce solar heating, and the top was covered with high-density PE film to minimize the effect of air convection. A solar power meter (Taiwan TES1333R, Taipei, Taiwan, China) was used to measure the variation in solar irradiance during the entire temperature measurement process, with an average solar irradiance of 430.74 W/m^2^ (Figure 4b). Matlab R2024a simulations were performed to calculate the cooling power under different convective heat transfer coefficients (hc = 0, 3, 6, 9, 12) at a solar irradiance of 1000 W/m^2^, resulting in a theoretical net cooling power for the 15CA coating of approximately 94.67 W/m^2^ (Figure 4c). Additionally, the temperature variations in the ambient temperature, the coated 15CA sample, and the uncoated Al substrate were monitored over time (Figure 4d). Comparing the temperature changes, it was found that the temperature of the 15CA-coated sample decreased by 2.6 °C relative to the ambient temperature (Figure 4e), and by 18.9 °C compared to the uncoated Al substrate (Figure 4f).

**Figure 5:**
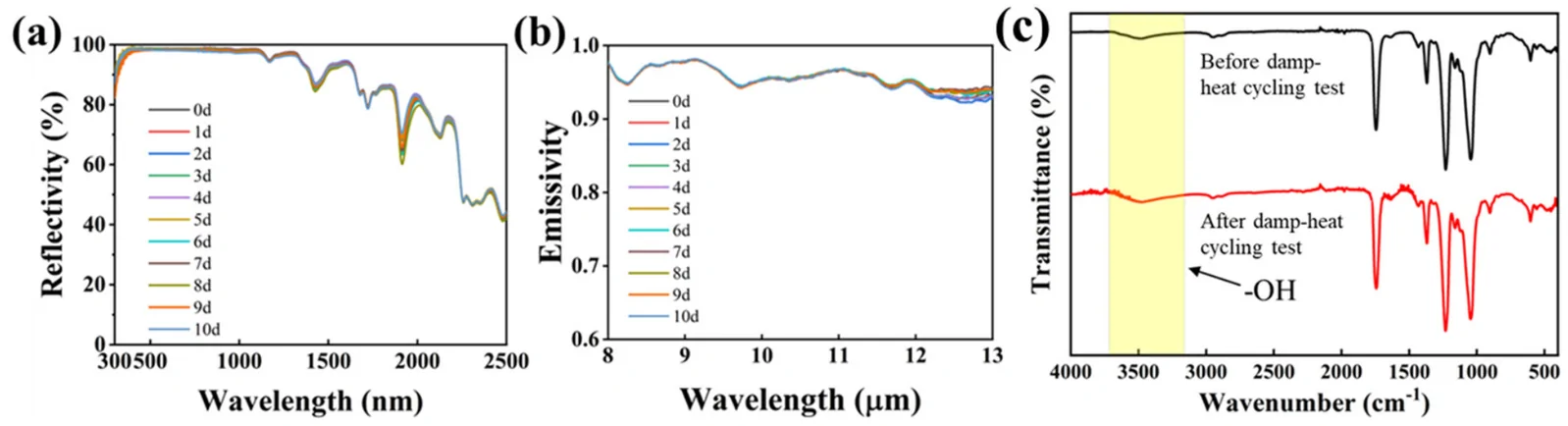
(**a**) Changes in the reflectance and (**b**) changes in the emissivity of the CA coating during the 10-day hot–humid test, and (**c**) infrared spectra of the CA coating before and after the hydrothermal cycling test.

The prepared coatings were subjected to hydrothermal cycling tests (conducted in accordance with the GJB 150.9A-2009 standard). According to the standard, 10 cycles are sufficient to reflect the potential effects of a hot–humid environment on the coatings. Therefore, the total test duration in this study was set to 10 cycles (240 h). To ensure the accuracy and timeliness of the test data, the coating samples were removed immediately after the completion of each cycle, and the optical performance tests were completed within 30 min. The test items included: solar reflectance (300–2500 nm) and infrared emissivity (8–13 μm). The changes in the optical performance of the coatings over the 10 cycles are shown in Figure 5a,b. We found that after 10 cycles, the 15CA coating showed no obvious change in appearance, and its optical performance declined slightly; nevertheless, the reflectance remained around 96%, and the emissivity remained above 0.96. The excellent performance of the cellulose acetate coating prepared by the NIPS method in hot–humid environments is mainly attributed to the stress-dispersion effect of its porous structure, the low moisture absorption resulting from acetylation modification, and the high thermal stability of the material itself. First, the cellulose acetate coating prepared by the NIPS method typically possesses a unique porous structure, which not only endows the material with a high specific surface area and good breathability, but also enables it to effectively disperse stress in hot–humid environments, thereby avoiding local deformation or structural damage caused by moisture absorption. In addition, after acetylation modification, the hydroxyl groups on the molecular chains of cellulose acetate are replaced by acetyl groups, and this chemical modification significantly reduces the hydrophilicity and moisture absorption of the material. The reduced moisture absorption allows the coating to maintain excellent dimensional stability and mechanical properties during the hydrothermal cycling tests, effectively suppressing the swelling, deformation, or degradation of surface properties caused by moisture absorption. Meanwhile, cellulose acetate exhibits high thermal stability and is not prone to thermal degradation or changes in its chemical structure during the hot–humid test, thus ensuring the long-term stability of its optical properties (such as reflectance and emissivity) and appearance.

The coatings after the hydrothermal cycling tests were characterized by infrared ATR spectroscopy. The results showed that a significant change occurred in the characteristic absorption peak of hydroxyl groups (-OH) at 320–3600 cm^−1^, specifically manifested as an increase in peak intensity and a broadening of the peak width (Figure 5c). This phenomenon is mainly attributed to the combination of free hydroxyl groups in the cellulose acetate molecular chains that were not completely acetylated with water molecules from the environment, forming stable intermolecular hydrogen bonds. These test results confirm that although acetylation modification significantly improves the hydrothermal resistance of the coating, a certain number of hydrophilic groups still exist on the material surface, causing the coating to exhibit moderate moisture absorption under hot–humid conditions.

## 4. Conclusions

This study fabricated CA coatings with distinct pore structure distributions. Experimental results demonstrate that pore architecture significantly influences the optical performance of coatings: the 15CA coating exhibited optimal characteristics, achieving an average solar reflectance of 96.56% in the solar spectral range (0.3–2.5 μm) and an emissivity of 0.96 within the atmospheric transparency window (8–13 μm). Comparative analysis between CA powder and the 15CA coating revealed that optimized porous structures enhance light scattering, thereby improving reflective performance. The 15CA coating, selected for its superior optical properties, underwent theoretical net cooling power calculations and outdoor cooling tests. Theoretical analysis evaluated radiative cooling power under varying non-radiative heat transfer coefficients (hc = 0, 3, 6, 9, 12 W/m^2^·K), incorporating parameters such as atmospheric emissivity, coating optical properties, solar irradiance (AM1.5G spectrum), and convective losses. This yielded a maximum theoretical net cooling power (P_net_) of 94.67 W/m^2^. In a 2 h outdoor test under solar irradiance of 430.74 W/m^2^, the 15CA-coated substrate demonstrated superior thermal management: 18.88 °C temperature reduction compared to uncoated substrates and 2.6 °C sub-ambient cooling relative to surrounding environmental temperature. The coating was subjected to a damp–heat cyclic test for 10 cycles; its reflectance was still maintained at around 96%, and its emittance remained above 0.96. These results validate the practical efficacy of pore-engineered CA coatings in radiative cooling applications.

## Data Availability

The original contributions presented in this study are included in the article. Further inquiries can be directed to the corresponding authors.
